# Development and validation of a structured questionnaire for assessing risk factors of medication non-adherence among pulmonary tuberculosis patients in Indonesia

**DOI:** 10.3389/fphar.2023.1257353

**Published:** 2024-01-16

**Authors:** Leonov Rianto, Ika Agustina, Sofa D. Alfian, Aulia Iskandarsyah, Ivan Surya Pradipta, Rizky Abdulah

**Affiliations:** ^1^ Department of Pharmacology and Clinical Pharmacy, Faculty of Pharmacy, Universitas Padjadjaran, Bandung, Indonesia; ^2^ IKIFA College of Health Science, Jakarta, Indonesia; ^3^ Center of Excellence for Pharmaceutical Care Innovation, Universitas Padjadjaran, Bandung, Indonesia; ^4^ Department of Clinical Psychology, Faculty of Psychology, Universitas Padjadjaran, Bandung, Indonesia

**Keywords:** tuberculosis, non-adherence, questionnaire development, questionnaire validation, SEM-PLS, prediction

## Abstract

**Background:** Medication non-adherence is a significant concern in tuberculosis (TB) treatment, requiring a precise understanding of the associated risk factors. However, there is a lack of appropriate means to assess the risk factors among TB patients in Indonesia, leading to the development and validation of a structured questionnaire for this purpose.

**Method:** This study unfolded in two distinct phases, namely, the first included questionnaire construction through framework development, item generation, item screening, and pretesting (in 50 patients). The second comprised questionnaire validation with 346 participants using confirmatory factor analysis (CFA) and structural equation modeling-partial least squares (SEM-PLS). Additionally, reliability testing was conducted using Cronbach’s alpha and composite reliability statistical techniques.

**Results:** In the development phase, 168 items were defined, consisting of sociodemographic characteristics (8 items) and risk factors for medication non-adherence (160 items). Expert evaluation reduced the number of items to 60, which decreased to 22 after performing a pilot study. Subsequent SEM-PLS modeling resulted in the identification of 14 valid items, representing five major risk factors, namely, socioeconomics (4 items), healthcare team (4 items), condition (3 items), therapy (2 items), and patient (1 item). Only condition-related factors were found to influence non-adherence, and all constructs showed good reliability based on Cronbach’s alpha (>0.6) and composite reliability (0.7) values.

**Conclusion:** The final 22 items that emerged from this rigorous process indicated a valid and robust questionnaire for assessing risk factors of medication non-adherence among pulmonary tuberculosis patients in Indonesia. The developed questionnaire was positioned to be a valuable tool for healthcare professionals, policymakers, and scientists in creating patient-centered strategies and interventions to address non-adherence.

## 1 Background

Tuberculosis (TB) is a significant public health concern, particularly in regions such as Asia and Africa, with the highest fatality rates emanating from infectious diseases worldwide (Zhu et al., 2022). In 2018, TB initiated greater mortality accounting for 1.5 million deaths, compared to HIV/AIDS (Harding, 2020). The complex, prolonged, and often poorly tolerated regimens for both drug-susceptible and resistant TB pose substantial challenges to treatment adherence (Alipanah et al., 2018; Pradipta et al., 2021; Pradipta et al., 2022a). Moreover, non-adherence to necessary medications increases the risk of negative outcomes, including treatment failure, elevated TB transmission, relapse, drug resistance emergence, as well as higher morbidity and mortality (Fang et al., 2019; Saha et al., 2022). Many patients fail to complete the full 6-month course of anti-TB medications, jeopardizing their health and contributing to the development of multidrug-resistant and extensively resistant. According to the World Health Organization (WHO), TB therapy adherence means the extent to which the prescribed pharmaceutical regimen is being followed. Several quantitative studies (El Sahly et al., 2004; Munro et al., 2007; Shargie and Lindtjørn, 2007) investigated risk variables linked to suboptimal treatment adherence, but only a few explored the relationship shared with socioeconomic factors. These sources showed that low education level, place of residence, financial constraints, comorbid chronic diseases, medication discontinuation, and anti-TB treatment frequency influence non-adherence (Alipanah et al., 2018; Zhu et al., 2022).

In the study conducted in rural and urban districts of the Democratic Republic of Timor-Leste, it was found that information about TB, its treatment, and the availability of incentives, such as transportation cost reimbursement or food support, positively influenced adherence (Ruru et al., 2018a). Four key determinants contribute to non-adherence, namely, structural (e.g., poverty and gender discrimination), social, and health service-related factors, as well as individual considerations (World Health Organization, 2003; Munro et al., 2007). An Indonesian study revealed that the most common reasons for non-adherence included patients feeling better, economic issues, and side effects of therapy. Other reasons were bad perceptions about the healthcare staff, treatment, and medication quality (Widjanarko et al., 2009). Recent investigations indicate the significance of socioeconomic challenges and the lack of adequate patient support in contributing to high rates of treatment discontinuation in Indonesia (Global, 2021). Effective adherence relies on social support, which may include the presence of a treatment observer and health education (Widjanarko et al., 2009; Ruru et al., 2018a; Pradipta et al., 2022b). Additional barriers to this consist of a preference for traditional medicine and economic and geographical problems (Ruru et al., 2018a; Pradipta et al., 2023).

Several existing questionnaires, such as the Morisky Medication 8-item Adherence Scale (MMAS-8) and MARS-5 Medication Adherence Report Scale-5 items (MARS-5) have been widely used to assess patient adherence to ongoing treatment (Lee et al., 2013; Rosyida, 2015; Naafi et al., 2016; Pradipta et al., 2020; Iranpour et al., 2022). However, their suitability for measuring non-adherence levels remains uncertain. There is no universally accepted gold standard questionnaire for evaluating non-adherence, specifically within the scope of TB. To address this gap, structural equation modeling-partial least squares (SEM-PLS) analysis was applied to develop a questionnaire that can be used to create a predictive model. The SEM-PLS approach comprised two distinct phases, i.e., the evaluation of measurement and structural models (Kono and Sato, 2022; Kori and Azmi, 2022).

Tuberculosis still poses a significant health challenge in Indonesia, necessitating interventions tailored to the diverse settings of the country (World Health Organization, 2003; Lestari et al., 2023). Therefore, this study presents a meticulously designed questionnaire for assessing the factors contributing to medication non-adherence among TB patients. Drawing inspiration from the five-dimensional framework established by WHO, the questionnaire was developed based on comprehensive systematic reviews and prior qualitative studies (DiMatteo, 2004; Munro et al., 2007). Importantly, before pilot testing, the initial development phase did not include direct patient input, instead, the primary focus was placed on capturing expert perspectives to refine item selection (World Health Organization, 2003). While the questionnaire yields statistically robust insights, its true value lies in practical applications. Considering the vast geographical and sociocultural differences in Indonesia, this tool is designed for flexible integration into various local contexts, facilitated through collaborations with local health entities (Lutge et al., 2014; Jimmy and Jose, 2011). Additionally, it is intended for use among TB patients in the early stages of treatment, capturing critical insights during this crucial period. The results can aid healthcare professionals in refining treatment adherence strategies and serve as a foundation for policymakers aiming to enhance TB management on a national scale (Stirratt et al., 2015).

## 2 Methods

### 2.1 Ethics approval

This study obtained approval from the ethics committees of Universitas Padjadjaran (No: 086/UN6. KEP/EC/2021), private hospitals (No: 1212/XIII/12/2020), and public hospitals (No: 13/KEPK-RSUPP/02/2021). Additionally, it was conducted in accordance with the Helsinki Declaration, and all participants provided informed consent.

### 2.2 Study design and sample size

The initial phase of questionnaire development constituted the engagement of a cohort of 50 patients, each with an extensive TB treatment regimen spanning a minimum of 6 months. This collective cohort participated in an inaugural assessment aimed at quantifying the efficacy of the measuring instrument in capturing the underlying construct. The construct validity assessment primarily focused on evaluating the ability of the questionnaire to measure the intended variables. Subsequently, a purposive sampling strategy was applied in the validation phase, targeting patients with a shorter TB treatment duration, ranging from one to 2 months.

For the validation phase, a representative sample was selected from the cohort of newly diagnosed patients in the Jakarta area between 2020 and 2021. Based on the Indonesian Health Profile report, published by the Ministry of Health, it was determined that Jakarta discovered a total of 28,125 cases in 2021 compared to the 24,274 recorded in 2020. This signified a discernible increment of 3,851 cases, which constituted the entire patient population scrutinized in this study.

The following two distinct methods were used to determine the optimal sample size: 1) The Krejcie and Morgan table in conjunction with the population parameters was deployed to obtain an optimal sample size ranging from 346 to 351 patients; 2) Alternatively, the Slovin formula, a well-established mathematical construct was applied for calculating sample size, with an error margin (e) of 5% (0.05). This included using the formula n = N/(1 + Ne^2), yielding a minimum sample size of 362 patients.

**FIGURE 1:**
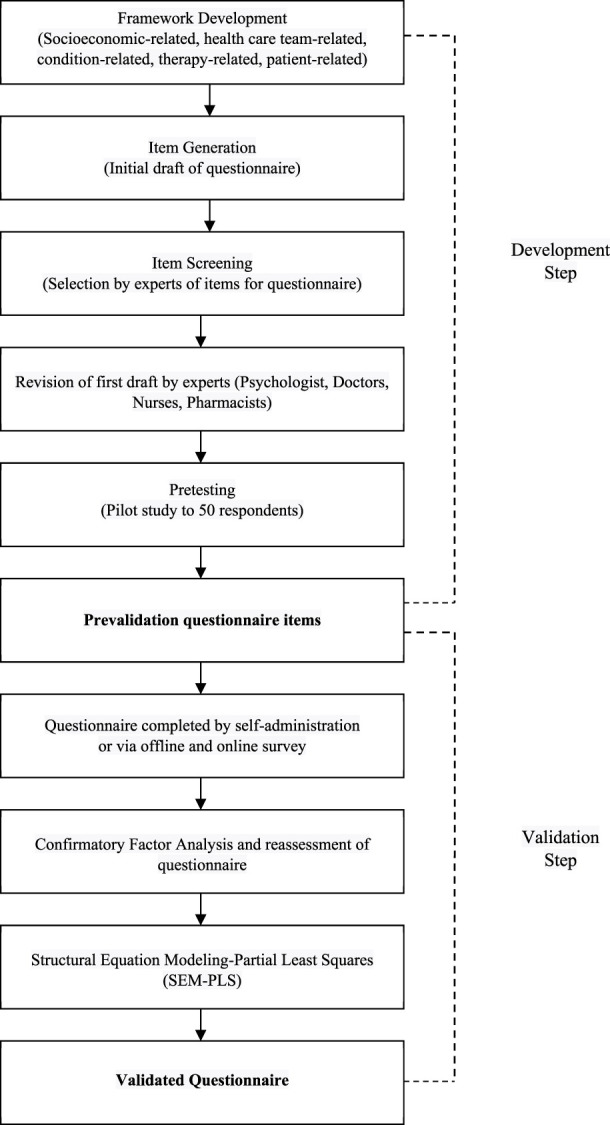
Flowchart of questionnaire development and validation.

In summary, the methodological framework determined a required sample size ranging from 346 to 362. Consequently, the comprehensive patient cohort for this study comprised 396 individuals, out of which 50 were actively engaged in the developmental phase and the remaining 346 were allocated to the validation stage. A flow diagram indicating the development and validation of the questionnaire is presented in Figure 1.

### 2.3 Development of questionnaire items

#### 2.3.1 Framework development

A systematic review and a qualitative study were initially conducted to identify relevant factors used to construct a questionnaire for predicting TB patient non-adherence. Subsequently, a framework that described the five factors influencing long-term medication adherence, namely, socioeconomic status, healthcare team, medical conditions, therapy, and patients, was adopted from the WHO (World Health Organization, 2003).

#### 2.3.2 Item generation

Building upon the previously established framework, questionnaire items representing each dimension or variable to be measured were developed. Although the framework primarily pertained to adherence, this study adapted all the obtained dimensions to the context of non-adherence. Five items created for each indicator in the variable were assessed by TB treatment experts and analyzed by psychologists.

#### 2.3.3 Item screening

A panel of experts, including a psychologist, three pulmonary specialists, two nurses, and three pharmacists, assessed the level of difficulty and adequacy of the questionnaire. This evaluation was based on qualitative study activities conducted before the questionnaire development. The experts participated in focus group discussions (FGD) regarding the factors influencing non-adherence and were selected according to the possession of at least 1 year of experience in TB medication. Specifically, psychologists were engaged in assessing the readability and comprehensibility of the items before the pretesting stage. Following this process, the initial selection of items was refined based on assigned the median total score. Items that received higher scores, as indicated by multiple experts, proceeded to the next stage.

#### 2.3.4 Pretesting (pilot study)

After the panel of experts conducted a content validity assessment, a pilot questionnaire was pretested on 50 respondents in December 2021, as recommended by healthcare professionals. Criteria for participant selection included a history of medication non-adherence, age 18 years or older, a minimum of high school education, and willingness to provide informed consent. Respondents were recruited during hospital visits, and each completed a paper copy of the questionnaire. Trained assistants reviewed the self-administered questionnaires on-site before being delivered to the study team.

### 2.4 Validation of questionnaire items

The pilot study produced prevalidated questionnaire items that showed statistical validity and could be applied in a comprehensive validation process using SEM-PLS. The entire validation phase was conducted from January to March 2022 and respondents were selected through a purposive sampling technique. Selection criteria included sensitive TB patients recently placed on medication (1–2 months), aged 18 years or older, with a minimum of high school education, and willing to sign an informed consent. Questionnaires were distributed in one public and six private hospitals, as well as nine community health centers in Jakarta. An online Google survey was used for remote respondents registered as patients at the designated study location.

#### 2.4.1 Confirmatory factor analysis (CFA)

Confirmatory factor analysis (CFA) is an integral component of SEM, valuable for the appropriateness of variable measurements concerning the number of factors. In CFA, factors can be considered as constructs, and this analysis represents an interdependence technique for determining the underlying structure in construct variables. High partial correlation in factor analysis holds practical and statistical significance, with the general rule of thumb suggesting values above 0.70 as conceptually valid (Lance and Vandenberg, 2002; Harrington, 2009; Brown and Moore, 2012). However, the Bartlett roundness test at a level of >0.05 indicates a sufficient correlation between construct variables for a single-factor analysis (Suhr, 2006; Hair et al., 2014a; Gatignon and Gatignon, 2014; Brown, 2015).

#### 2.4.2 Structural equation modeling-partial least squares (SEM-PLS)

SEM is a statistical model that describes the relationships among several variables (Hair et al., 2014b). During the calculation process, SEM simultaneously examines the structural relationships expressed through a series of equations resembling multiple regression equations. These equations elucidate all the interconnections between analyzed constructs, comprising both dependent and independent variables. Constructs are unobservable and cannot be represented by numerous variables compared to those representing factors in CFA. Moreover, PLS-SEM is a causal-predictive method of SEM that stresses prediction in estimating statistical models aimed at providing causal explanations (Hair et al., 2019). In this study, each item used an ordinal Likert scale for measurement, with five potential response levels. Indicators with ordinal responses from at least four categories may be interpreted as intervals, or at the very least, as continuous variables. No two indicators for a construct must have the same scale type, and scale values need not be normalized (Hair et al., 2014b). The utility of a questionnaire as a study instrument is evaluated using the validity and reliability method. Validity refers to the extent to which observations accurately record the examined variables. Meanwhile, reliability relates to the consistency of observations, often determined by whether two (or more) observers or the same observers, monitoring the same event on successive occasions, reach similar conclusions (Sekaran and Bougie, 2016).

### 2.5 Statistical analysis

Data analysis was performed using SPSS Statistics for Windows (version 24.0) and SMART-PLS software (version 3.0) in an SEM-PLS environment. In the pretesting phase, bivariate Pearson correlation statistical analysis was applied to determine the validity of the items sorted by experts. Pearson correlation measures the relationship between observations from a population with two variants (bivariate), normally distributed. With the participation of 50 patients, items indicating a Pearson correlation value exceeding 0.278 proceeded to the validation phase, which used SEM. Besides, SEM-PLS incorporates a measurement model that evaluates the relationship between indicators and their latent variables, automatically presenting the factor load as an indicator of the validity of a factor or latent variable (Ghozali, 2014). Regarding the validity limits, indicators with factor loadings between 0.40 and 0.70 are considered for removal only when the scenario tends to enhance the composite reliability score. However, content validity factors must be considered during the elimination of these indicators. Indicators with factor loading values below 0.40 should be removed, and those between 0.40 and 0.70 may be retained supposing their presence does not adversely affect the average variance extracted (AVE) gain or composite reliability. In this study, the validity limit value used was 0.40, considering the applicable terms and conditions, as well as the significance of the coefficient at a 5% level (Hair et al., 2021). Composite reliability and Cronbach’s alpha were applied to assess the reliability of the study instruments. While these two methods use distinct calculation methodologies, both reveal the level of reliability for each latent or constructed variable. The minimum value required for optimal reliability is 0.60, with higher values indicating greater reliability (Hair et al., 2021). Computation analysis and validity-reliability testing were performed using SEM-PLS and SmartPLS 3 software (Hair et al., 2019). The value of composite reliability was assessed to test the reliability of each indicator on a variable, and >0.70 was considered the benchmark for high reliability. Specifically, reliability is essential for ensuring the precision and accuracy of measurements. Reliability testing was conducted by examining the value of Cronbach’s alpha to determine whether the data obtained from the instrument showed adequate internal consistency. Note that a study instrument is considered reliable once the Cronbach’s alpha value is >0.60 (Chin, 1998; Hair et al., 2014a; Ghozali, 2016).

## 3 Results

### 3.1 Development of questionnaire items

#### 3.1.1 Framework development

**FIGURE 2:**
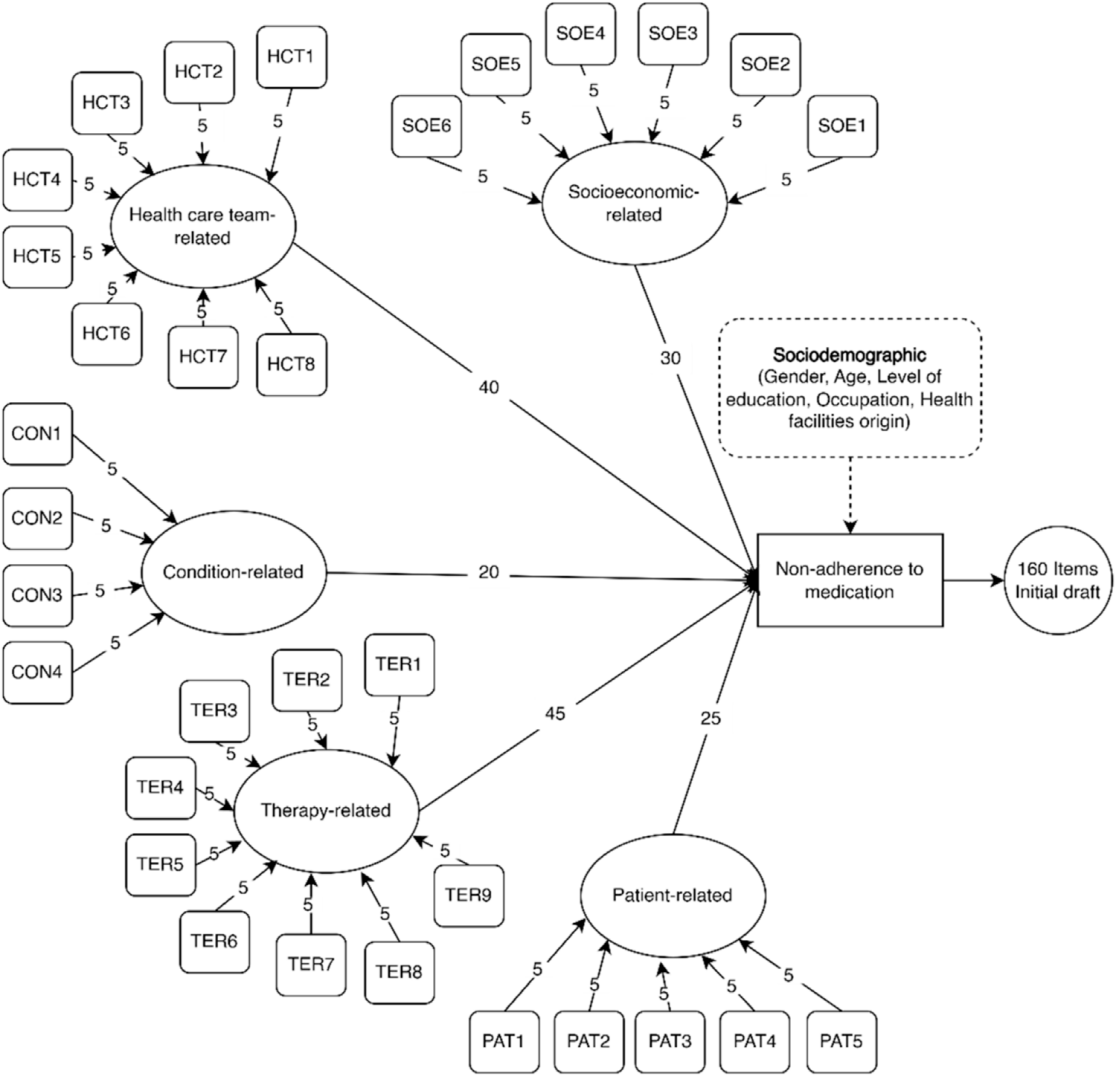
Framework development for questionnaire items.

The five-dimensional framework of WHO for adherence was adapted into the concept of non-adherence in this study. These five dimensions comprised the various causes and risk factors associated with non-adherence, based on the results of systematic reviews and qualitative studies conducted previously. Each dimension had specific indicators used for measuring its impact on medication non-adherence. A total of 32 indicators were successfully generated, with each contributing five items. As shown in Figure 2, the dimensions were as follows: socioeconomic (6 indicators; 30 items), healthcare team (8 indicators; 40 items), conditions (4 indicators; 20 items), therapy (9 indicators; 45 items), and patients (5 indicators; 25 items). This resulted in the generation of a total of 160 items in the subsequent stage.

#### 3.1.2 Item generation

The items generated for the questionnaire were adapted to local settings and divided into six main sections, as presented in Table 1. These included Demographic factors (5 items) which examined demographic and socioeconomic characteristics. Socioeconomic-related factors (30 items) constituting considerations such as economic priorities. In developing countries, patients with low socioeconomic status often face the challenge of balancing competing priorities. These competing priorities might require allocating limited resources to meet the needs of other family members, such as the children or parents catered for (Killewo, 2002; World Health Organization, 2003; Diniawati and Wibowo, 2018; Mahara et al., 2018). Healthcare team-related factors (40 items) explored the effects of the patient-provider relationship, and more investigations are needed concerning the impact of the healthcare team and system-related factors on non-adherence. While an excellent patient-provider relationship can increase adherence several factors have a negative effect. These are comprised of underdeveloped healthcare services, inadequate or nonexistent reimbursement by health insurance plans, poor drug distribution systems, and a lack of knowledge and training among healthcare providers in managing chronic diseases (World Health Organization, 2003; Do Peterson et al., 2012; Gugssa Boru et al., 2017). Condition-related factors (20 items) included demands, symptoms, and disease-specific issues targeted by healthcare professionals. Several conditional factors, such as patient geography and health status, influenced their willingness to complete medication (World Health Organization, 2003; Shargie and Lindtjørn, 2007; Tadesse et al., 2013; Woimo et al., 2017; Ruru et al., 2018b). Therapy-related factors (45 items) consisted of the main barriers to adherence found in intervention studies, such as dosing frequency and side effects. Collaboration between pharmaceutical companies, health professionals, and researchers is essential to address this issue. Health systems play an essential role in minimizing the impact of side effects (World Health Organization, 2003; Do Peterson et al., 2012; Gugssa Boru et al., 2017; Heuvelings et al., 2017). Patient-related factors (25 items) examined the primary barriers to compliance as described in the reviewed literature, namely, a lack of information and self-management skills, difficulties with motivation and self-efficacy, and inadequate support for behavioral change (Morisky et al., 1990; Chambers et al., 2010; Gugssa Boru et al., 2017). These barriers are specifically relevant for interventions aimed at changing habits and lifestyles, as well as influencing drug use. The WHO recognizes the need to support patient self-management efforts, and many researchers are working to develop, enhance, and disseminate self-management guidelines (World Health Organization, 2003; Gough and Kaufman, 2011; Van Den Boogaard et al., 2012; Chowdhury et al., 2015; Sahile et al., 2018).

**TABLE 1:** Details of questionnaire sections on sociodemographic and all factors.

Section	No. of items	Concept measured	Response options
Sociodemographic	5	1) Gender	Closed-ended, multiple-choice
		2) Age	
		3) Education level	
		4) Occupation	
		5) Health facilities origin	
Socioeconomic-related	30	1) Availability of social support from fellow patients	1 = Strongly disagree 2 = Disagree
		2) Bad perception of disease (Communities)	3 = Not sure
		3) Environmental support	4 = Agree
		4) Fear of infecting families	5 = Strongly Agree
		5) Getting financial and logistical assistance	
		6) Social and family support	
Healthcare team-related	40	1) Availability of disease education by health workers	1 = Strongly disagree 2 = Disagree
		2) Availability of information and education	3 = Not sure
		3) Communication effectiveness	4 = Agree
		4) Lack of health facility services	5 = Strongly Agree
		5) Limitations of treatment services	
		6) Limited and inaccurate information	
		7) Negative prognosis of health professionals	
		8) Quality of service from healthcare professionals	
Condition-related	20	1) Availability of facilities and affordable health facilities	1 = Strongly disagree 2 = Disagree
		2) Dirty and unhealthy work environment	3 = Not sure
		3) Long distance to health facilities	4 = Agree
		4) Deteriorating and uncontrolled patient conditions	5 = Strongly Agree
Therapy-related	45	1) Comorbidity	1 = Strongly disagree
		2) Drug resistance	2 = Disagree
		3) Impact of treatment on activities	3 = Not sure
		4) Impact of treatment on health conditions	4 = Agree
		5) Lower pill burden	5 = Strongly Agree
		6) More efficient drug preparations	
		7) Relapse/retreatment	
		8) Supporting therapy	
		9) Treatment side effects	
Patient-related	25	1) Stigma against disease (Patient)	1 = Strongly disagree
		2) Motivation to live	2 = Disagree
		3) Motivation for adhering to treatment	3 = Not sure
		4) Negative perceptions of disease and treatment	4 = Agree
		5) Self-vulnerability	5 = Strongly Agree

This section consisted of choices on a scale from one to five, with response categories ranging from “strongly disagree” to “strongly agree” for each question. Following the results of the item selection by experts, a total of 60 questionnaire items were used in the pilot study. At this stage, the expectation was to obtain a questionnaire containing a more streamlined set of items to facilitate measurement with fewer items during validation.

#### 3.1.3 Item screening

In this phase, the initial selection of 160 items was reduced to 60, based on a median total score of 2.0 for each. Items selected by more than two experts proceeded to the next stage.

#### 3.1.4 Pretesting (pilot study)

The results of face validity obtained during the pilot study featuring 50 respondents reduced the number of questionnaire items from 60 to 22. The response rate was 100% (50/50 participants), with respondents requiring an average of 15 min to complete the questionnaire. The validity of each item is presented in the Pearson correlation column in Table 2. Considering the 50 respondents (N) and a significance level of 0.05, the minimum Pearson correlation value was 0.278. Therefore, 22 items exhibited Pearson correlation values exceeding 0.2732, denoted by * or ** in the Pearson correlation column of the output table. As a result, 38 items were considered invalid, while 22 were validated.

**TABLE 2:** The results of questionnaire validation through expert review and statistical analysis.

No.	Items	Expert choice	Pearson correlation	Sig. (2-Tailed)	N
1	SCA11	3	0.015	0.916	50
2	SCA12	5	0.081	0.575	50
**3**	**SCA21**	**9**	0.614**	**0**	**50**
**4**	**SCA23**	**9**	0.519**	**0**	**50**
**5**	**SCA24**	**3**	0.701**	**0**	**50**
6	SCA31	3	0.156	0.278	50
7	SVA13	3	0.09	0.532	50
8	SVA14	3	−0.024	0.869	50
9	SVA22	5	0.194	0.177	50
10	SVA23	3	−0.099	0.495	50
11	SVA31	6	0.008	0.957	50
12	SVA34	3	0.031	0.832	50
**13**	**SVA41**	**8**	0.552**	**0**	**50**
14	SVA44	3	−0.1	0.49	50
15	SVA51	3	−0.122	0.397	50
16	BNA11	6	0.112	0.439	50
17	BNA14	3	−0.164	0.255	50
18	BNA21	4	0.215	0.134	50
**19**	**BNA31**	**6**	0.370**	**0.008**	**50**
**20**	**BNA34**	**5**	0.633**	**0**	**50**
21	BNA41	4	−0.013	0.93	50
22	BNA43	3	0.114	0.432	50
23	BNA51	3	0.109	0.449	50
24	BNA52	3	0.215	0.134	50
25	BNA61	4	0.029	0.844	50
26	BNA63	4	0.048	0.74	50
27	BRA14	4	0.171	0.235	50
**28**	**BRA21**	**6**	0.586**	**0**	**50**
29	BRA33	3	−0.033	0.821	50
30	BRA35	4	0.089	0.54	50
31	BRA41	4	0.097	0.503	50
32	BRA44	3	−0.129	0.371	50
33	BRA51	3	0.148	0.305	50
34	BRA55	4	0.073	0.612	50
35	BRA65	4	0.15	0.299	50
**36**	**SEA11**	**8**	0.501**	**0**	**50**
**37**	**SEA15**	**7**	0.554**	**0**	**50**
**38**	**SEA23**	**9**	0.464**	**0.001**	**50**
39	SEA31	4	0.064	0.661	50
40	SEA33	3	−0.107	0.461	50
41	SEA45	4	0.159	0.271	50
**42**	**CAA11**	**7**	0.597**	**0**	**50**
**43**	**CAA12**	**5**	0.690**	**0**	**50**
**44**	**CAA13**	**7**	0.579**	**0**	**50**
**45**	**CAA14**	**4**	0.750**	**0**	**50**
**46**	**CAA15**	**4**	0.406**	**0.003**	**50**
47	CAA22	4	0.072	0.617	50
48	CAA23	4	−0.133	0.357	50
49	CAA33	6	−0.097	0.502	50
50	CAA35	3	0.118	0.416	50
**51**	**CAA41**	**5**	0.721**	**0**	**50**
**52**	**CAA42**	**5**	0.590**	**0**	**50**
**53**	**CAA43**	**6**	0.702**	**0**	**50**
**54**	**CAA44**	**6**	0.648**	**0**	**50**
**55**	**CAA45**	**4**	0.506**	**0**	**50**
56	CAA51	4	0.202	0.16	50
57	CAA61	7	−0.027	0.854	50
58	CAA65	4	0.071	0.625	50
**59**	**CAA71**	**7**	0.567**	**0**	**50**
**60**	**CAA72**	**6**	0.434**	**0.002**	**50**

The bold values in Table 2 serve as highlights to indicate items with significance levels above 1% and above 5%, helping to differentiate which items are considered valid and can be progressed to the next stage.

Specifically, ** represents significance above 1%, while * represents significance above 5%. In this context, the value 0.278, marked with a *, signifies a significance level of 5%, indicating that this item is valid for further consideration. Conversely, the value 0.354, marked with **, signifies a significance level of 1%, further emphasizing its validity for progression to the next stage.

#### 3.1.5 Prevalidated questionnaire items

The 22 questionnaire items identified during the face validity assessment were administered to a total of 346 sensitive TB patients as respondents. None of the patients from the pilot study were included in the validation phase. Table 3 presents an overview of selected items and themes that successfully passed face validity.

**TABLE 3:** Twenty two-item and theme validation study.

Factor	No.	ID	Theme
Socioeconomic-related	1	SCA21	Families understand TB disease suffered
	2	SCA23	Cutlery/drinks are separated from those of family members
	3	SCA24	Cutlery, clothes, and items are washed separately
	4	SEA23	Financial and moral support needed from the family
	5	CAA45	Talks to the family about medical conditions and the burden
Healthcare team-related	1	SVA41	Takes medication before the test results come out
	2	CAA41	Undergoes treatment after receiving an explanation of the procedure
	3	CAA42	Knows the side effects and therapy of drugs
	4	CAA43	The team of health workers continues to communicate during treatment
	5	CAA44	Speaks with the doctor/nurse because the information is not understood
Condition-related	1	CAA11	Takes alternative medicine to aid healing
	2	CAA12	Takes other drugs to relieve side effects of treatment
	3	CAA13	Does light exercise regularly
	4	CAA14	Maintains the diet
	5	CAA15	Consumes herbs to promote breathing
Therapy-related	1	BNA31	The amount of medication taken has decreased with the start of treatment
	2	BNA34	No more injections when coming to health facilities
	3	CAA71	Excited to undergo treatment once the number of drugs is reduced
	4	CAA72	It feels better to take medicine than to have an injection
Patient-related	1	BRA21	Side effects decrease after a long course of treatment
	2	SEA11	Needs support to recover and undergo treatment
	3	SEA15	Needs information and education for treatment

**FIGURE 3:**
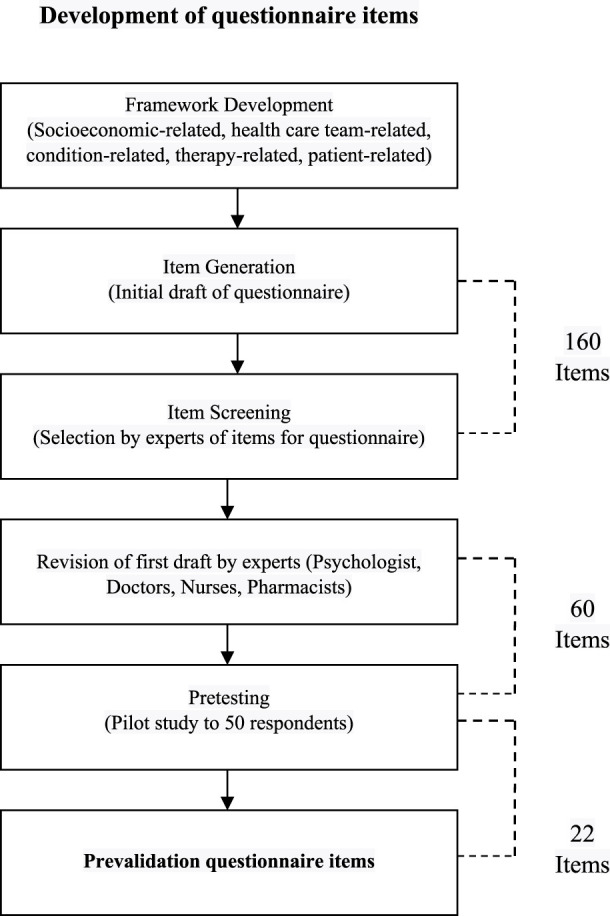
Process and results of questionnaire development.

Overall, the results of the questionnaire development phase can be seen in Figure 3.

### 3.2 Validation of questionnaire items

#### 3.2.1 Sociodemographic details of respondents

Table 4 presents the demographic characteristics of the 346 respondents who participated in this study. The most common age range found was 20–29 years (24.28%), with a mean of 39.71 (±10.71) years. Most of the respondents were male (54.91%). The educational background was predominantly high school (82.37%), while some (17.34%) completed tertiary education. A significant proportion was unemployed (39.30%) and a small percentage was health workers (0.76%). A substantial number came from private hospitals (38.15%), which was justifiable because these hospitals served as referral centers for pulmonary diseases in Jakarta.

**TABLE 4:** Sociodemographic characteristics of respondents.

Sociodemographic characteristic	n (%)
Gender	
Male	190 (54.91%)
Female	155 (44.80%)
Rather not to say	1 (0.29%)
Age (years)	
<20	23 (6.65%)
20–29	84 (24.28%)
30–39	75 (21.68%)
40–49	64 (18.50%)
50–59	53 (15.32%)
>60	47 (13.58%)
Level of Education	
Highschool	285 (82.37%)
Undergraduate	59 (17.05%)
Postgraduate	1 (0.29%)
Rather not to say	1 (0.29%)
Occupation	
Employed	124 (35.84%)
Enterpreneur	84 (24.28%)
Unemployed	136 (39.30%)
Rather not to say	2 (0.58%)
Health Facilities Origin	
Community Health Center	104 (30.06%)
Private Hospital	132 (38.15%)
Public Hospital	110 (31.79%)
Medication Status	
Complete	255 (73.70%)
Incomplete	91 (26.30%)
Reasons for Incomplete Medication	
Not Evaluated/Moved	75 (82.42%)
Failed/Not Completed	16 (17.58%)

#### 3.2.2 Confirmatory factor analysis (CFA)

Table 5 shows the results for each measured factor, as each item in all the factors was tested for factor loading to identify the influence of each item.

**TABLE 5:** Analysis of all factors.

Factor	Cronbach’s alpha	Composite reliability	AVE
Socioeconomic-related	0.29	0.60**	0.43
Healthcare Team-related	0.47	0.67**	0.40
Condition-related	0.70**	0.80**	0.46
Therapy-related	0.66**	0.80**	0.50**
Patient-related	−1.09	0.26**	0.60**

The asterisk (*) in Cronbach’s Alpha and Composite Reliability signifies a high level of statistical significance or strong validity. In simpler terms, it indicates that the measurements or constructs being assessed are reliable and consistent for the analysis or research being conducted.

Standardized factor loadings were expected to exceed 0.70, but factor loadings in the 0.40–0.70 range could be evaluated. The evaluation conducted featured content validity by considering the impact exerted on the AVE gain and composite reliability. A higher factor loading value signified greater validity of the construct measurement (Hair et al., 2014b). Factor loadings must be statistically significant, with t values exceeding 1.96 for a 5% significance level (Hair et al., 2014b). To measure convergent validity, the AVE was used, with a threshold value of 0.50. A higher AVE value indicated more information obtained from the latent and reflected similarity in the latent construct (Hair et al., 2014b). Composite reliability and Cronbach’s alpha ought to have a value of at least 0.70, although a minimum of 0.60 is acceptable for exploratory studies (Hair et al., 2014b). Regarding cross-loading, each indicator should exhibit a stronger correlation with its construct than other constructs, indicating discriminant validity. This empirical standard ensures that a measured construct is distinct from other constructs (Hair et al., 2014b).

##### 3.2.2.1 Socioeconomic-related factors

The composite reliability value for socioeconomic-related factors was >0.7, confirming the suitability of the data for factor analysis. Based on commonality, one item (SEA23) had a very low factor loading and was excluded from further analysis, as it would not correlate with other items representing socioeconomic-related factors. Additionally, one item (SCA21) had the lowest factor loading (0.52) but was retained in the analysis because a factor loading >0.4 was considered the minimum acceptable value. The other three items (SCA23, SCA24, CAA45) met the criteria for reliability and were retained. In summary, only SEA23 was excluded and four items (SCA23, SCA24, SEA23, and CAA45) were considered reliable for socioeconomic-related factors (Table 6).

**TABLE 6:** Factor loading of 22-items.

Factor	Items	Factor loading
Socioeconomic-related	SCA21	0.52*
	SCA23	0.75**
	SCA24	0.75**
	SEA23	−0.58
	CAA45	0.62*
Healthcare team-related factors	SVA41	−0.26
	CAA41	0.65*
	CAA42	0.63*
	CAA43	0.72**
	CAA44	0.76**
Condition-related factors	CAA11	0.78**
	CAA12	0.67*
	CAA13	0.62*
	CAA14	0.48*
	CAA15	0.78**
Therapy-related factors	BNA31	0.72**
	BNA34	0.55*
	CAA71	0.79**
	CAA72	0.74**
Patient-related factors	BRA21	0.71**
	SEA11	−0.84
	SEA15	0.77**

In the context of loading factors in Structural Equation Modeling (SEM), the asterisk (*) typically indicates that the loading factor has achieved a high level of statistical significance or strong validity. In simpler terms, it suggests that the measurement variable has a strong influence on the factor or construct being measured in the SEM model.

##### 3.2.2.2 Healthcare team-related factors

The composite reliability value for healthcare team-related factors also exceeded 0.7, indicating data suitability for factor analysis. One item (SVA41) showed a very low factor loading and was excluded from further analysis, as it would not correlate with others representing healthcare team-related factors. Additionally, two items (CAA41 and CAA42) had relatively low factor loadings but were maintained in the analysis due to their factor loadings exceeding the minimum acceptable value of 0.4. The other two items (CAA43 and CAA44) met the reliability criteria and were retained. In summary, only SVA41 was excluded and CAA41, CAA42, CAA43, and CAA44 were considered reliable for healthcare team-related factors (Table 6).

##### 3.2.2.3 Condition-related factors

The composite reliability value for condition-related factors was >0.7, indicating data suitability for factor analysis. No item had low factor loading, consequently all were included in further analysis. One item (CAA14) had a low factor loading of 0.48 but was maintained in the analysis due to being greater than the minimum acceptable value of 0.4. The remaining four items (CAA11, CAA12, CAA13, and CAA15) met the reliability criteria and were retained. In summary, all five items were considered reliable for condition-related factors (Table 6).

##### 3.2.2.4 Therapy-related factors

The composite reliability value for therapy-related factors was >0.7, indicating that the data were suitable for factor analysis. No item had low factor loading, consequently all were included in further analysis. One item (BNA34) showed the lowest factor loading (0.55) but was retained, as it exceeded the minimum acceptable value of 0.4. The other three items (BNA31, CAA71, and CAA72) met the reliability criteria and were retained. All four items were considered reliable for therapy-related factors (Table 6).

##### 3.2.2.5 Patient-related factors

The composite reliability for patient-related factors was >0.7, indicating data suitability for factor analysis. One item (SEA11) had a very low factor loading and was excluded from further analysis, as it would not correlate with other items representing patient-related factors. The remaining two items (BRA21 and SEA15) were deemed acceptable and retained patient-related factors (Table 6), hence only SEA11 was excluded.

#### 3.2.3 Structural equation modeling-partial least square (SEM-PLS)

**FIGURE 4:**
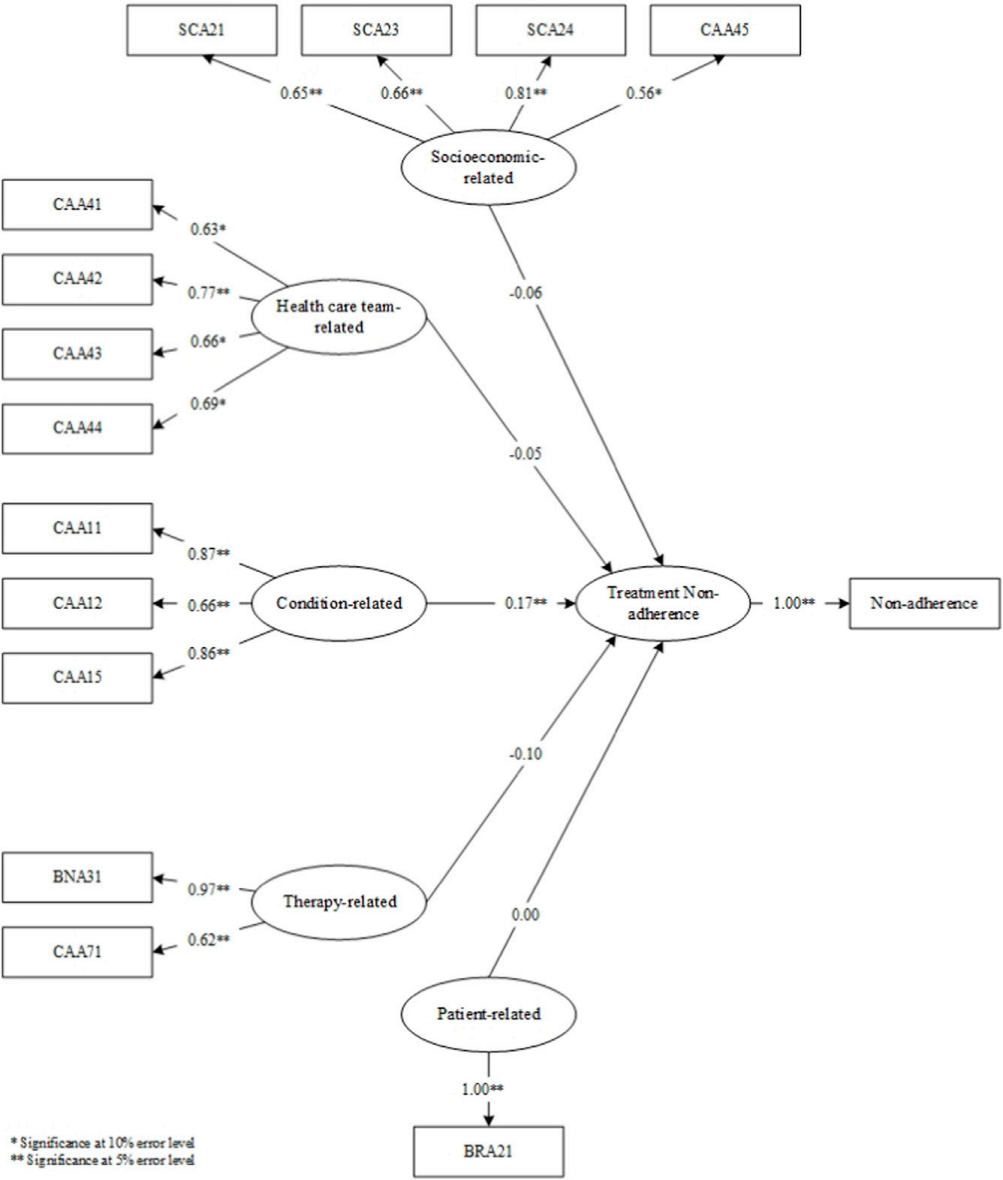
SEM-PLS modeling.

The results of the questionnaires at the validation stage determined the items that proceeded to the SEM-PLS modeling stage (Figure 4). Upon model simulation, differences emerged between valid items in factor loadings at the analysis stage and factor loadings on SEM. In the analysis stage, SEA23, SVA41, and SEA11 were deemed invalid. In the simulated SEM-PLS model, SEA23, SVA41, CAA13, CAA14, BNA34, CAA72, SEA11, and SEA15 were excluded. The three items, including SEA23, SVA41, and SEA11, remained invalid in both factor analysis and SEM despite sharing similarities. CAA13, CAA14, BNA34, CAA72, and SEA15 which were valid in the factor analysis became invalid in SEM. This showed that SEM examined the effect of each item on the factor measured, and the influence of the factor on non-adherence. Consequently, SEM yielded more invalid items compared to factor analysis.

In the simulated SEM-PLS model, only one factor, namely, condition, significantly influenced non-adherence. This indicated why all condition-related factor items remained entirely valid at the analysis stage. However, the therapy-related factors had no impact on non-adherence in the SEM-PLS model. In comparison to the other four, condition-related factors significantly influenced patient non-adherence to medication.

Following the SEM-PLS modeling process, 14 valid items remained across the five factors, including those related to socioeconomics (4 items; SCA21, SCA23, SCA24, CAA45), healthcare team (4 items; CAA41, CAA42, CAA43, CAA44), medical condition (3 items; CAA11, CAA12, CAA15), therapy (2 items; BNA31, CAA71), and patients (1 item; BRA21). Only condition-related factors significantly influenced non-adherence, and as indicated in Table 7, the constructs developed were reliable. The reliability test conducted in PLS applied Cronbach’s alpha and composite reliability techniques. Cronbach’s alpha measures the lower limit of the reliability value of a construct, while composite reliability estimates the actual value. Composite reliability is considered better at estimating the internal consistency of a construct. Moreover, the rule of thumb used for the composite reliability value indicated >0.7, and the obtained Cronbach’s alpha value exceeded 0.6 (Chin, 1998; Ghozali, 2016), signifying that all constructs had good reliability.

**TABLE 7:** Analysis of all factors after SEM-PLS modeling.

Factor	Cronbach’s alpha	Composite reliability	AVE
Socioeconomic-related	0.62**	0.77**	0.46
Healthcare Team-related	0.65**	0.78**	0.48
Condition-related	0.72**	0.84**	0.64**
Therapy-related	0.60**	0.79**	0.66**
Patient-related	1.00**	1.00**	1.00**

The asterisk (*) in Cronbach's Alpha and Composite Reliability signifies a high level of statistical significance or strong validity. In simpler terms, it indicates that the measurements or constructs being assessed are reliable and consistent for the analysis or research being conducted.

**FIGURE 5:**
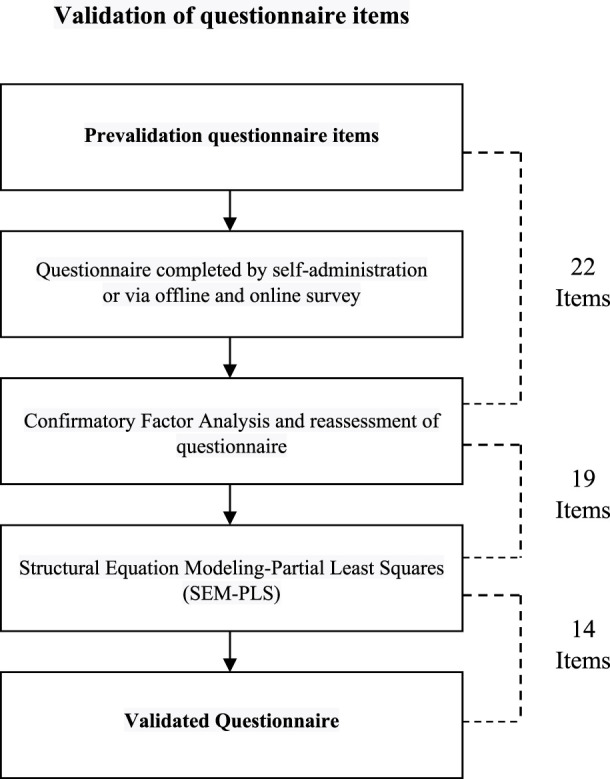
Process and results of questionnaire development.

Overall, the results of the questionnaire validation phase can be seen in Figure 5.

## 4 Discussion

Following a rigorous validation process featuring nine experts, including a psychologist, three pulmonary specialists, two nurses, and three pharmacists, as well as 50 patients from two health facilities, 22 items were obtained for the final draft of the questionnaire. The questionnaire was tested using 346 TB patients who had only been on medication for 1–2 months. Additionally, it was distributed offline and online in accordance with ethical research agreements. The results of this study showed the influence of each item on the factors examined.

The questionnaire was suitably developed to address the complexities of the diverse regions in Indonesia and the unique healthcare challenges encountered. Indonesia has been reported to show significant regional disparities in healthcare infrastructure, patient attitudes, and socioeconomic influences (Erawati and Andriany, 2022; Siswantining et al., 2020; Pratiwi et al., 2020). Considering these disparities, the questionnaire was designed to be adaptable and relevant across different areas of the country, but some regional customization was required before the broad implementation (Mahmudiono and Laksono, 2021). In the initial phase of item development, the questionnaire was created based on evidence gathered from systematic reviews and qualitative studies rather than direct patient engagement (Bowden and Fox-Rushby, 2003). This choice was influenced by studies suggesting that expert-driven item generation often provides a more structured foundation for pilot testing (Collins, 2003). The questionnaire was designed to assess medication non-adherence factors in TB patients and intended for use across public and private healthcare settings in Indonesia, providing actionable insights for healthcare professionals. The insights provided by this questionnaire (Mekonnen and Azagew, 2018; Vaughan et al., 2019) were expected to assist policymakers, medical practitioners, and scientists in enhancing healthcare delivery and patient adherence to improve treatment outcomes (DiMatteo, 2004; Departemen Kesehatan, 2018; Kemenkes, 2020).

This study identified socioeconomic, healthcare team, condition, therapy, and patient-related issues as the five main factors contributing to medication non-adherence in TB patients. One item in each of the socioeconomic, healthcare team, and patient-related factors did not exert statistically significant effects, while all items in both condition and therapy were found to have significant impacts. Additionally, the factor loading value considered for each item was ≥0.40. The influence of each factor on the possibility of patient non-adherence was examined as presented in Figure 4. The analysis results showed that only condition-related factors significantly influenced medication non-adherence.

This study applied methods similar to those used in previous investigations conducted in Sabah, Malaysia (Guad et al., 2021). While several methods were replicated, the primary difference could be found in the analytical method. This study combined CFA with SEM-PLS, but other sources commonly used a single method, such as CFA or Exploratory Factor Analysis (EFA) (Deng et al., 2017; Soh et al., 2018; Gunawan et al., 2021). EFA is mostly used in cases where initial information is lacking or when hypotheses must be derived from a set of indicators, leading to the creation of variables from these indicators (Gorsuch, 1988; Fabrigar et al., 1999; Cudeck, 2000; Suhr, 2006; Fabrigar and Wegener, 2011; Hooper, 2012). During the analytical process, CFA was conducted because the indicators and variables were known. Besides, SEM-PLS is a relatively less utilized method for developing questionnaires, primarily due to its prevalence in investigations focused on predictive modeling (Brown, 2015). The combination of CFA and SEM-PLS was deployed to elucidate the capability of the developed and validated questionnaire to measure the impact of indicators on variables or dimensions and the effect of each variable on non-adherence (Brown and Moore, 2012; Hair et al., 2019; Kono and Sato, 2022). Through SEM-PLS analysis, this questionnaire was used to construct a predictive model for predicting TB patient non-adherence at the onset of treatment.

In the aspect of statistical analysis, this study applied robust methodological tools, specifically CFA and SEM-PLS, to examine the empirical results (Hair et al., 2019; Erawati and Andriany, 2022). This methodological choice was based on the predictive potential of the carefully developed and validated questionnaire. The CFA and SEM-PLS techniques not only clarified the complex causal pathways underlying the observed phenomena but could also forecast future trends (Luies et al., 2017). These methodologies synergistically facilitated a comprehensive examination of the relationships within the model, enabling predictions and enhancing the understanding of variable interactions (Collins et al., 2015; Dessalegn et al., 2016).

The applied methodologies were intentionally selected due to certain considerations. Despite other approaches, such as the regression technique, being valid and widely utilized, the distinctive focus of this study necessitated a non-traditional approach (Zhu et al., 2022; Do Peterson et al., 2012; Chowdhury et al., 2015; Erawati and Andriany, 2022). The adopted approach created a distinct path suitably tailored to the inherent intricacies and nuances of the study question. In summary, the utilization of CFA and SEM-PLS represented a streamlined approach. Additionally, the predictive potential of the model constructed from the questionnaire resonated strongly with the applied methods. These statistical tools impart explanatory power and the invaluable ability to predict future trends.

The questionnaire served as a foundation for constructing a predictive model for non-adherence. Furthermore, the score of each item in it offered valuable insights into the influence exerted on non-adherence. Theoretically, this study provided an overview of the steps and procedures for developing and validating questionnaires used to assess non-adherence in TB patients as well as those suffering from other diseases. The questionnaire could be practically tested in various provinces across Indonesia or Southeast Asia, supporting healthcare providers in delivering appropriate services to patients at risk of non-adherence. However, this study is currently limited to measuring non-adherence in TB patients due to the unique demographic conditions in Indonesia.

## 5 Conclusion

A structured questionnaire was successfully developed to assess medication non-adherence among TB patients in Indonesia. The final 22 items that emerged from this rigorous process indicated a valid and robust questionnaire for assessing risk factors of medication non-adherence among pulmonary tuberculosis patients in Indonesia. The developed questionnaire was positioned to be a valuable tool for healthcare professionals, policymakers, and scientists in creating patient-centered strategies and interventions to address non-adherence.

## Data Availability

The raw data supporting the conclusion of this article will be made available by the authors, without undue reservation.
